# A rare case report of an solitary neurofibroma in postcricoid region of hypopharynx

**DOI:** 10.1097/MD.0000000000024017

**Published:** 2021-01-08

**Authors:** Dongjie Li, Kai Niu, Tingting Yuan, Wei Zhu, Xin Wang, Wanzhong Yin

**Affiliations:** a Department of Otolaryngology-Head & Neck Surgery; b Department of Radiology, First Hospital of Jilin University, Changchun, Jilin Province, People's Republic of China.

**Keywords:** solitary neurofibroma, postcricoid region, hypopharynx, transoral microsurgery, supporting laryngoscope

## Abstract

**Rationale:**

Postcricoid neurofibroma is an extremely rare hypopharynx tumor that can be challenging in both diagnosis and treatment. This case sheds light on the possibility of treatment with transoral microsurgery before pursuing open cervical incisions.

**Patient concerns:**

: A 43-year-old man presented with a four months history of a persistent foreign body sensation and mild dysphagia. Indirect and direct laryngoscopy at admission revealed a round and smooth submucosal mass in the postcricoid region.

**Diagnosis:**

A laryngeal enhanced computed tomography and laryngoscopy suggested that the tumor located in hypopharynx, with clear boundary and slightly strengthened edge. A supporting laryngoscopy surgery was performed under general anesthesia and a biopsy confirmed solitary neurofibroma of the postcricoid region.

**Interventions:**

The tumor was successfully resected en bloc transorally through supporting laryngoscope, and obviated the need for open cervical surgery and tracheostomy.

**Outcomes:**

The patient recovered well without any intraoperative or postoperative complication and was discharged from hospital 2 days after surgery. There was no recurrence after 6 months follow-up.

**Lessons:**

Postcricoid neurofibroma is an extremely rare hypopharynx tumor that can be diagnostically challenging. To the best of our knowledge, this is the first case reported of solitary neurofibroma originating from the postcricoid region of the hypopharynx and was surgically removed with transoral surgery through supporting laryngoscope.

## 1 Introduction

Neurofibromas are well known as a kind of benign peripheral nerve sheath tumors and consists of axons, Schwann cells, perineurial cells, fibroblasts, and mast cells.^[1]^ There are totally 3 subtypes of neurofibroma: localized, plexiform, and diffuse. Although a clear definition for solitary neurofibroma is still lacking, it is considered a hyperplastic hamartomatous malformation rather than a neoplastic disease.^[2]^

Space occupying lesions in the postcricoid region of hypopharynx are extremely rare. According to some reported articles, hemangioma, lymphangioma, solitary fibrous tumor, sarcoidosis, leiomyosarcima, adenoid cystic carcinoma and squamous cell carcinoma in postcricoid region have been reported^[3–9]^. The treatment of postcricoid mass is based on the pathology and functional significance of the lesion, and includes minimally invasive surgery and open cervial surgery based comprehensive treatment^[10]^. To the best of our knowledge, we present the first case of solitary neurofibroma ever reported in the English literature originating from the postcricoid region of the hypopharynx. It was surgically removed through supporting laryngoscope, eliminating the need for open cervical surgery.

## 2 Case report

**Figure 1:**
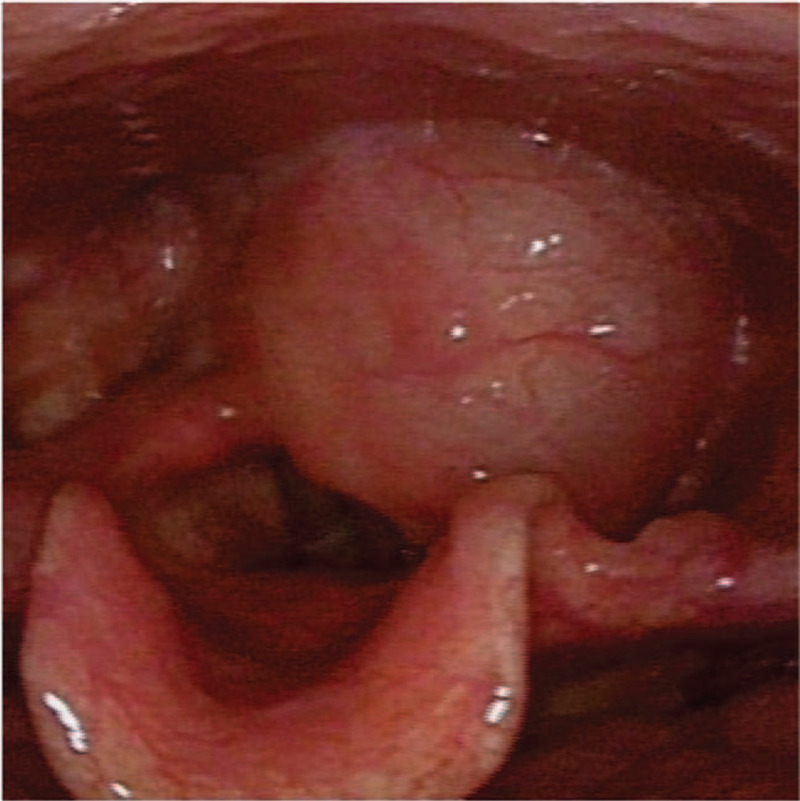
Laryngoscopy demonstrated a round and smooth submucosal mass in the postcricoid region.

**Figure 2:**
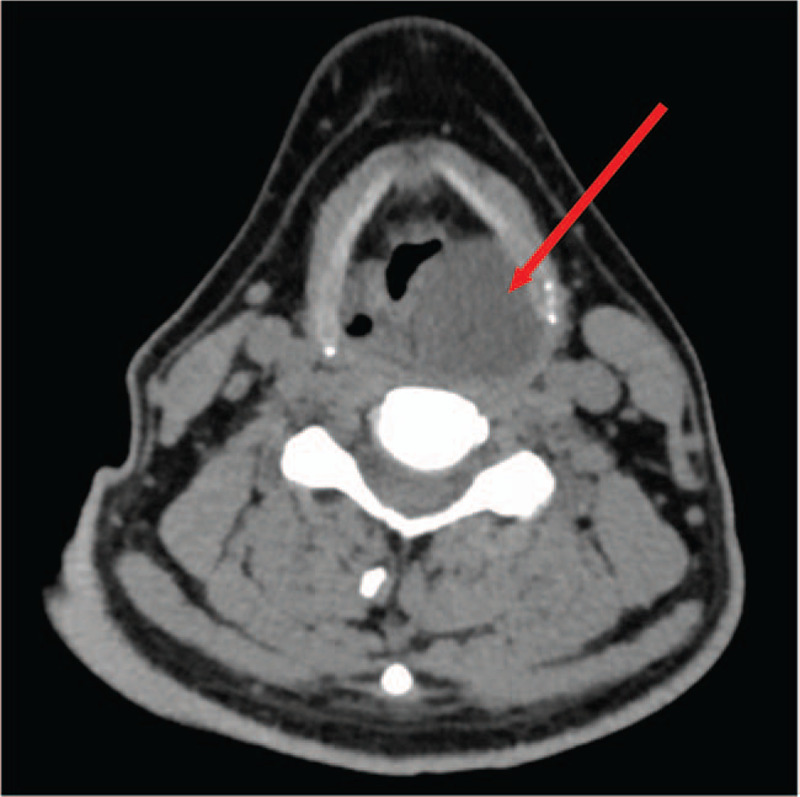
A laryngeal enhanced CT revealed a space occupying lesion of approximately 4.5cm × 3.0 cm × 2.0 cm in size located in postcricoid area.

A 43-year-old man presented at the our Department of Otolaryngology-Head & Neck Surgery, with a four months history of a persistent foreign body sensation and mild dysphagia, but he denied weight loss and dyspnea. He had been unsuccessfully treated with medication for pharyngitis before finally being evaluated by an otolaryngologist. His history was otherwise unremarkable. Indirect laryngoscopy at admission revealed a round and smooth submucosal mass in the postcricoid region and further laryngoscopy confirmed it (Fig. 1). The vocal folds’ mobility was preserved and there was no clinically palpable lymphadenopathy, no other abnormalities were detected (in particular, skin changes such as café au lait spots, axillary freckling, or subcutaneous nodules). A laryngeal enhanced CT suggested that the lesion was approximately 4.5 cm × 3.0 cm × 2.0 cm in size located in hypopharynx, with clear boundary and slightly strengthened edge, and the CT value was about 20HU (Fig. 2). All these features suggested a cystic mass.

**Figure 3:**
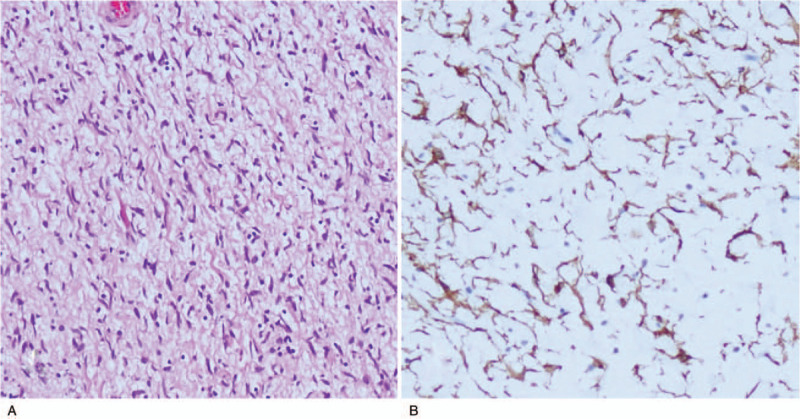
Histology of the tumor. A: photomicrograph showed bundles of fibroblast-like cells with abundant collagen. HE stain × 100. B: Immunohistochemical staining for CD34 showed that most tumor cells were diffusely positive.

**Figure 4:**
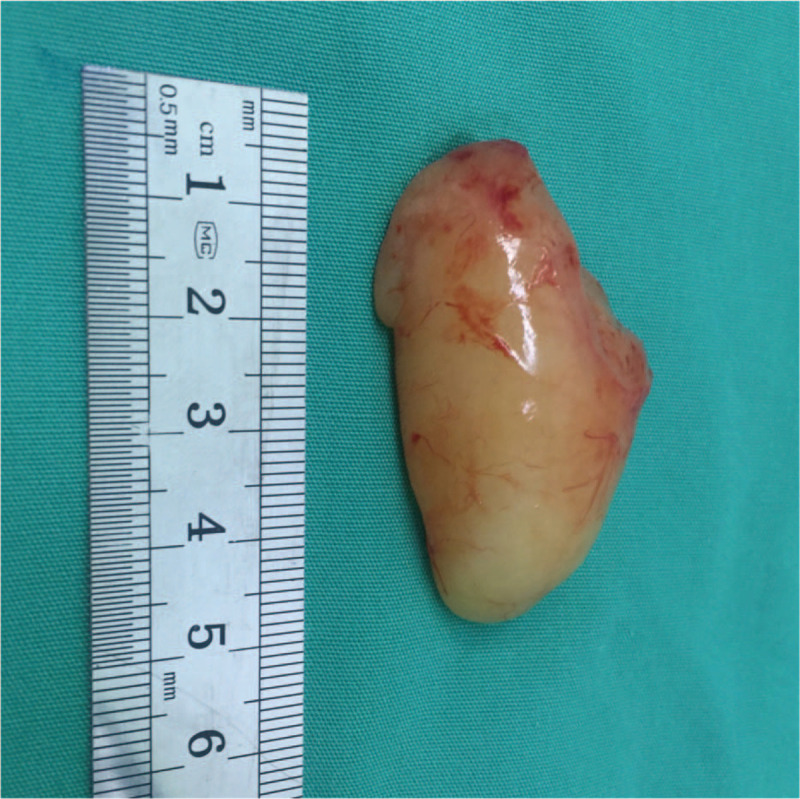
After surgical resection of the postcricoid neurofibroma, the tumor appeared yellowish, oval-shaped and was about 4.5cm × 2.5cm × 1.5 cm in size.

A transoral microsurgery through supporting laryngoscopy was performed under general anesthesia. After fully exposing the postcricoid region, a biopsy through a mucosal incision was performed. Submucosal dissection exposed the tumor and allowed gradual detachment of it from the surrounding mucosa by microforceps. The procedure revealed a yellowish mass with oval-shaped appearance. The microscopic histological and immunohistochemical stains confirmed a solitary neurofibroma (Fig. 3). Further detachment of tumor was successful on account of being well-circumscribed and easy to dissect from the surrounding tissues. The tumor was en bloc resected through supporting laryngoscope. The tumor was about 4.5cm × 2.5cm × 1.5 cm in size (Fig. 4). Then the incision was sutured with mucosa-to mucosa opposition.

The patient stopped fasting and recovered well without any intraoperative or postoperative complication and was discharged from hospital 2 days after surgery. A laryngoscope examination and CT scan were performed 6 month later with no signs of recurrence.

## 3 Discussion

Neurofibroma is a well-circumscribed, non-encapsulated, intraneural growing tumor, associated with the potential to involve the adjacent tissues.^[11]^ Its formation is due to the excessive proliferation of Schwann's cells, fibroblasts, neurons, and perineural cells.^[12]^ Although neurofibroma may occur in any possible anatomic location of myelinated nerves, very few reports have demonstrated neurofibromas in hypopharynx. To the best of our knowledge, solitary neurofibroma of the postcricoid region has not been previously reported so far in peer-reviewed English journals.

Solitary neurofibroma is not associated with Neurofibromatosis-1 (NF1), and appears to affect teenagers and young adults without gender preference.^[13]^ The clinical manifestations of solitary neurofibroma are not specific and alter according to their locations. Clinically, patients may be asymptomatic for a long period of time and symptoms are frequently due to compression of the surrounding organs. In our case, the patient was a male adult presenting with persistent foreign body sensation and mild dysphagia due to tumor compression on digestive tract. The patient did not have obstruction of the respiratory tract such as hoarseness, stridor, and apnea.

On radiological imaging, predominantly, solitary neurofibroma manifests as a well-delineated smooth margin solid fusiform mass.^[14]^ Among all the reported cases, two thirds of the solitary neurofibromas exhibited uneven contrast enhancement.^[15,16]^ Preoperative imaging is usually insufficient to establish the diagnosis, histopathology is still the golden standard of making a definite diagnosis. In general, neurofibroma can be differentiated from schwannoma, solitary neurofibroma as it may be well-bordered without encapsulation,^[13,17]^ while schwannomas are often encapsulated and the capsule can be delineated. From histopathological aspect, with the aid of immunohistochemical stains, S-100 protein has been used classically as a marker in the differential diagnosis of neurofibromas from schwannomas. The immunoreactivity of S-100 protein is usually higher in schwannomas than neurofibromas. Recently, the application of CD34 has been reported to differentiate neurofibromas from schwannomas, neurofibromas are strongly positive for CD34 in contrast to most schwannomas. In our case, CD34 was diffusely positive for most tumor cells, and fibroblast-like cells showed especially strong positive signals, and the tumor was well-bordered without encapsulation. All of these characteristics suggest the formal diagnosis of neurofibroma.

The differential diagnosis of postcricoid masses is the same as those in other regions of the hypopharynx which includes squamous cell carcinoma and other kinds of less common entities such as adenocarcinomas, lymphomas, and sarcomas. The diagnosis may be based on history and examination followed by biopsy of the tissue involved.

Surgical removal is the preferred treatment option for neurofibroma. However, the tumor size and its vascularization often make surgical resection challenging.^[18]^ The preoperative histological evidence can influence the surgical approach, conservative or aggressive.^[19]^ When preoperative biopsy is not available and the diagnosis is uncertain, or there is concern for hemorrhagic complications, surgical removal may be indicated. In the past, lesions of postcricoid region were mostly approached through open cervical incisions.^[20]^ Recently, many reports now favor transoral microsurgery, which spares an external incision and improves functional outcomes despite repeat debulking of tumor. With transoral approaches, a much lower tracheostomy rate, shorter time to decannulation after being placed and a shorter hospitalization were observed compared with cervical open surgery,^[21,22]^ furthermore, functional outcomes in terms of voice and swallowing are favorable. Minimally invasive management can be achieved with endoscopic resection. Exposure is a known limiting factor for transoral microsurgery; adequate exposure for transoral microsurgery mandates a direct line of sight for both visualization and instrumentation.^[23]^ The postcricoid region presents a unique surgical challenge because it is positioned between 2 rigid structures - the posterior surface of the cricoid cartilage and C5 and C6 vertebral bodies. This decreases the flexibility required to strengthen visualization when approaching from a transoral route. Additionally, the current microexcision instruments available have limitations in operating in this location. Furthermore, severe symptomatic upper airway obstruction may require a tracheostomy. In presenting case, we successfully detached and resected tumor en bloc through supporting laryngoscope. This is the first case reported of solitary neurofibroma originating from the postcricoid region of the hypopharynx that was surgically removed with transoral approach through supporting laryngoscope, avoiding the need for open surgery and tracheostomy. Solitary neurofibromas have a good prognosis, with rare instances of haemorrhage, malignant changes and local recurrences after excision.^[24]^

In summary, postcricoid neurofibroma is an extremely rare hypopharynx tumor that can be challenging for both diagnosis and treatment. This case sheds light on the possibility of treatment with transoral microsurgery before pursuing open cervical incisions.

## Author contributions

**Investigation:** Tingting Yuan.

**Resources:** Kai Niu.

**Supervision:** Xin Wang.

**Validation:** Wanzhong Yin.

**Writing – original draft:** Dongjie Li.

**Writing – review & editing:** Wei Zhu.
